# Clinical Pattern of Dermatological Diseases in Geriatric Patients: A Cross-Sectional Study From Central India

**DOI:** 10.7759/cureus.78468

**Published:** 2025-02-03

**Authors:** Venus N Sadhwani, Nidhi J Vithalani, Akanksha P Dani, Ramesh J Hasani

**Affiliations:** 1 Department of Dermatology, Employee State Insurance Corporation (ESIC) Hospital, Nagpur, IND; 2 Divison of Clinical Research and Training, St. John's Research Institute, Banglore, IND; 3 Department of Community Medicine, All India Institute of Medical Sciences, Nagpur, IND; 4 Department of Critical Care Medicine, Arihant Multispeciality Hospital, Nagpur, IND

**Keywords:** ageing, geriatric, geriatric dermatology, skin diseases, systemic diseases

## Abstract

Introduction

Geriatric dermatology, a growing subspecialty within dermatology, addresses the unique skin health challenges faced by the elderly population. The aging process stimulates a wide range of dermatological changes, which can be classified into intrinsic and extrinsic factors. The interplay between systemic diseases and skin health in geriatric patients highlights the importance of a multidisciplinary approach to diagnosis and treatment. Thus, the present study was planned in order to explore the patterns of dermatological diseases in geriatric patients as well as to determine the association of dermatological diseases with systemic diseases.

Materials and methods

This was a hospital-based cross-sectional design to examine the clinical patterns of dermatological diseases in geriatric patients. Conducted over 18 months (2022-2023) in the Department of Dermatology, Venereology, and Leprology at a tertiary care center in Central India, the study included patients aged 60 years and above who attended the outpatient department (OPD). A total of 482 patients were enrolled using consecutive sampling, where all eligible patients during the study period who provided informed consent were included.

Results

Eczemas were the most prevalent condition (100 patients, 20.74%), with a higher prevalence in males (67, 23.8%) than in females (33, 16.5%; p = 0.065). Fungal infections were also more frequent in males (44, 15.6%) compared to females (16, 8.0%; p = 0.014), while contact dermatitis was significantly higher in females (31, 15.5%; p < 0.001). Age-related trends showed eczemas peaking in the 80-89 age group (eight, 28.6%), while malignancies were more prevalent in the 70-79 age group (four, 3%; p = 0.031). Systemic diseases such as hypertension (86, 17.8%) and diabetes mellitus (50, 10.4%) were linked to dermatological conditions, with eczemas (15, 17.4%) and fungal infections (12, 24% in diabetics) being particularly associated.

Conclusion

This study highlights the significant interplay between dermatological conditions and systemic diseases in geriatric patients, with notable gender and age-specific variations. Integrated, multidisciplinary approaches are essential to address the unique dermatological and systemic health needs of the elderly population.

## Introduction

Geriatric dermatology, a growing subspecialty within dermatology, addresses the unique skin health challenges faced by the elderly population [1]. The aging process stimulates a wide range of dermatological changes, which can be classified into intrinsic and extrinsic factors. Intrinsic aging, a natural process influenced by genetic and physiological factors, is common across all individuals and is characterized by structural and functional changes in the skin, such as thinning of the epidermis, loss of elasticity, and reduced sebaceous gland activity [2]. Extrinsic aging results from environmental exposures, including ultraviolet (UV) radiation, smoking, and other pollutants, which exacerbate skin damage through oxidative stress and inflammation [3].

Elderly individuals are susceptible to dermatological diseases due to the accumulative impact of physiological aging and the increased prevalence of systemic illnesses associated with advancing age. The decline in mobility, frequent polypharmacy, and heightened susceptibility to chronic diseases contribute to this vulnerability. Conditions such as hypertension (HTN), diabetes mellitus (DM), human immunodeficiency virus (HIV), ischemic heart disease (IHD), dementia, and chronic hepatic or renal dysfunction can aggravate skin health conditions [4]. These systemic diseases impair vascular function, hinder immune responses, and compromise the wear and tear mechanisms of tissue, leading to delayed wound healing and, thus, a higher incidence of cutaneous infections and complications [4].

The interplay between systemic diseases and skin health in geriatric patients highlights the importance of a multidisciplinary approach to diagnosis and treatment. For instance, DM often manifests as xerosis, pruritus, or diabetic dermopathy, while chronic renal disease may present with pallor, pruritus, or calciphylaxis [1,4,5]. Similarly, cardiovascular diseases can cause stasis dermatitis and leg ulcers due to compromised vascular efficiency. These dermatological conditions not only impact the physical well-being of elderly patients but also affect their mental health, contributing to diminished quality of life [6].

Environmental factors play a significant role in exacerbating dermatological issues in the elderly. UV radiation, the primary cause of extrinsic aging, accelerates the degradation of collagen and elastin fibers in the dermis, leading to photoaging and increased risk of skin malignancies such as basal cell carcinoma, squamous cell carcinoma, and melanoma [3,7]. Smoking further contributes to skin aging by reducing oxygen supply and promoting free radical damage, while exposure to pollutants can trigger inflammatory skin disorders such as eczema or psoriasis [8,9].

Understanding the clinical patterns of dermatological diseases in geriatric patients requires a broad evaluation of intrinsic and extrinsic aging factors, systemic comorbidities, and environmental exposures. This knowledge is crucial for developing targeted prevention and management strategies, ultimately improving the overall health and quality of life for elderly individuals. Thus, the present study aims to analyze the prevalence and patterns of dermatological diseases among geriatric patients and explore their potential role in systemic diseases.

## Materials and methods

Study design

This was a hospital-based cross-sectional study where data was collected once from the patients.

Study setting

The study was conducted in the Department of Dermatology, Venereology, and Leprology of a tertiary care center located in central India.

Study duration

The study was conducted for 18 months, from 2022 to 2023.

Study population

All the geriatric patients, i.e., patients above 60 years of age attending the OPD of Dermatology, Venereology, and Leprology of a tertiary care center in central India, were considered potential populations for the study.

Sampling technique

Time frame sampling was done, i.e., all the patients fulfilling the inclusion criteria of the study and attending the OPD of tertiary care center during the study period of 18 months were enrolled in the study with their consent. A total of 482 patients were enrolled consecutively at the end of the data collection period.

Inclusion and exclusion criteria

Inclusion criteria included all new patients aged 60 years and above attending outpatient (geriatric clinic) in the Department of Dermatology, Venereology, and Leprology. “New patient” refers to patients aged 60 years and above who have not taken medicines for any dermatological conditions in the past six months OR patients aged 60 years and above who have visited the dermatology OPD of the institute for the first time. Patients who had not given consent or were willing for examination were excluded.

Ethical consideration

The study proceeded following the acquisition of ethical approval from the Institutional Review Board (Eth.Com/411). Data collected from patients on paper-based questionnaires are then entered into Microsoft Excel (Microsoft® Corp., Redmond, WA) and kept in a password-protected computer. Filled questionnaires were handled only by principal investigators and co-investigators and will be preserved for three years in lock and key.

Study procedure

Following obtaining written informed consent, the patient's medical history was documented, and a comprehensive examination encompassing general, systemic, and cutaneous aspects was conducted. All observed cutaneous and mucosal lesions were documented in the structured and validated questionnaire. Specific classification or nomenclature is used as per standard guidelines for the cutaneous and mucosal lesion documentation. Relevant investigations, including a basic hemogram, liver function tests, kidney function tests, fasting and postprandial blood sugar levels, lipid profile, and certain specialized tests (e.g., potassium hydroxide (KOH)), were conducted. Assessment of slit skin smears was conducted for leprosy patients. Biopsy is conducted when required. Systematic diseases were confirmed by history taking.

Data analysis

Data were analyzed using SPSS software version 24.0 (IBM SPSS Statistics for Windows, IBM Corp., Armonk, NY). Categorical data were analyzed in the form of frequency and percentages, and associations were found using non-parametric tests such as the chi-squared test and Fisher’s exact test.

## Results

The distribution of dermatological diseases among geriatric patients shows gender-specific differences. Among the total 482 patients (282 males and 200 females), eczema was the most prevalent condition, affecting 100 (20.74%) patients overall, with a higher proportion in males (67, 23.8%) compared to females (33, 16.5%; p = 0.065). Fungal infections were significantly more common in males (44, 15.6%) than in females (16, 8.0%; p = 0.014). Conversely, contact dermatitis was significantly more prevalent in females (31, 15.5%) than in males (19, 6.7%; p < 0.001).

Conditions like papulosquamous disorders, xerosis, and viral infections showed no significant gender difference, affecting both genders similarly (p > 0.05). Senile pruritus was more frequent in males (19, 6.7%) compared to females (six, 3.0%; p = 0.691). Other conditions, such as Hansen’s disease, photodermatoses, and bacterial infections, were distributed without significant gender variation (p > 0.05). Less common conditions like urticaria, fissured soles, vitiligo, and vesiculobullous disorders showed minimal gender-related differences. Rare diseases, such as malignancy, connective tissue disorders, and lichen amyloidosis, also exhibited no significant gender bias (Table 1).

**Table 1:** Distribution of dermatological diseases according to gender of geriatric patients

Diseases	Total		Male		Female		p-value
	N = 482	%	N = 282	%	N = 200	%	
Eczemas	100	20.74	67	23.8	33	16.5	0.065
Fungal infections	60	12.44	44	15.6	16	8.0	0.014
Contact dermatitis	50	10.37	19	6.7	31	15.5	<0.001
Papulosquamous disorders	47	9.75	29	10.3	18	9.0	0.518
Xerosis	40	8.29	25	8.9	15	7.5	0.612
Viral infections	32	6.63	17	6.0	15	7.5	0.506
Senile pruritus	25	5.18	19	6.7	6	3.0	0.691
Hansen’s disease	19	3.94	11	3.9	8	4.0	0.941
Photodermatoses	15	3.11	8	2.8	7	3.5	0.668
Bacterial infections	13	2.69	6	2.1	7	3.5	0.351
Urticaria	12	2.48	6	2.1	6	3.0	0.535
Fissured soles	12	2.48	6	2.1	6	3.0	0.535
Vitiligo	11	2.28	5	1.8	6	3	0.350
Vesiculobullous disorder	10	2.07	4	1.4	6	3.0	0.193
Adverse drug reaction	6	1.24	2	0.7	4	2.0	0.204
Scabies	6	1.24	4	1.4	2	1.0	1.00
Pruritus due to systemic diseases	6	1.24	4	1.4	2	1.0	0.071
Malignancy	5	1.03	2	0.7	3	1.5	0.408
Connective tissue disorder	3	0.62	0	-	3	1.5	0.071
Lichen amyloidosis	3	0.62	1	0..4	2	1.0	0.572
Granuloma annulare	2	0.41	1	0.4	1	0.5	1.00
Keloid	2	0.41	1	0.4	1	0.5	1.00
Black hairy tongue	1	0.20	1	0.4	0	-	0.413
Actinic porokeratosis	1	0.20	0	-	1	0.5	0.413
Lichen sclerosus et atrophicus	1	0.20	0	-	1	0.5	0.413

The distribution of diseases across different age groups (60-69 years, 70-79 years, and 80-89 years) shows variations in prevalence for specific conditions. Among the elderly population, eczema was the most common disease, affecting 100 (20.74%) patients overall, with the highest prevalence in the 80-89 age group (eight, 28.6%, p = 0.508). Fungal infections followed, affecting 60 (12.44%) patients of the total population, with a relatively even distribution across age groups (p = 0.679). Contact dermatitis was more common in the 70-79 and 80-89 age groups (18, 13.4% and four, 14.3%, respectively; p = 0.248). Other prevalent conditions include papulosquamous disorders (47, 9.75%) and xerosis (40, 8.29%), both distributed without significant differences by age. Less common conditions, such as Hansen’s disease (19, 3.94%) and bacterial infections (13, 2.69%), showed similar prevalence across age groups, while senile pruritus (20, 5.18%) increased with age. Urticaria, fissured soles, and vitiligo were relatively rare and evenly distributed. Significant differences were noted for malignancy, which was more prevalent in the 70-79 age group (four, 3.0%, p = 0.031). Rare conditions like lichen sclerosus et atrophicus and actinic porokeratosis were almost negligible (Table 2).

**Table 2:** Distribution of dermatological diseases according to age of geriatric patients

Diseases	Age group								p-value
	N	%	60-69 N = 320		70-79 N = 134		80-89 N = 28		
			N	%	N	%	N	%	
Eczemas	100	20.74	67	20.9	25	18.7	8	28.6	0.508
Fungal infections	60	12.44	41	12.8	17	12.7	2	7.1	0.679
Contact dermatitis	50	10.37	28	8.8	18	13.4	4	14.3	0.248
Papulosquamous disorders	47	9.75	35	10.9	11	8.2	1	3.6	0.358
Xerosis	40	8.29	26	8.1	12	9.0	2	7.1	0.933
Viral infections	32	6.63	24	7.5	7	5.2	1	3.6	0.564
Senile pruritus	25	5.18	13	4..1	9	6.7	3	10.7	0.202
Hansen’s disease	19	3.94	13	4.1	4	3.0	2	7.1	0.584
Photodermatoses	15	3.11	10	3.1	3	2.2	2	7.1	0.401
Bacterial infections	13	2.69	8	2.5	4	3.0	1	3.6	0.907
Urticaria	12	2.48	11	3.4	1	0.7	0	-	0.167
Fissured soles	12	2.48	9	2.8	3	2.2	0	-	0.642
Vitiligo	11	2.28	8	2.8	3	2.2	0	-	0.697
Vesiculobullous disorder	10	2.07	6	1.9	3	2.2	1	3.6	0.822
Adverse drug reaction	6	1.24	5	1.6	1	0.7	0	-	0.645
Scabies	6	1.24	5	1.6	1	0.7	0	-	0.642
Pruritus due to systemic diseases	6	1.24	2	0.6	4	3.0	0	-	0.098
Malignancy	5	1.03	1	0.3	4	3.0	0	-	0.031*
Connective tissue disorder	3	0.62	2	0.6	1	0.7	0	-	0.900
Lichen amyloidosis	3	0.62	2	0.6	1	0.7	-	-	0.901
Granuloma annulare	2	0.41	1	0.3	1	0.7	-	-	0.758
Keloid	2	0.41	1	0.3	1	0.7	-	-	0.758
Black hairy tongue	1	0.20	1	0.3	0	-	0	-	0.777
Actinic porokeratosis	1	0.20	1	0.3	0	-	0	-	0.777
Lichen sclerosus et atrophicus	1	0.20	0	-	0	-	1	3.6	0.058

**Figure 1:**
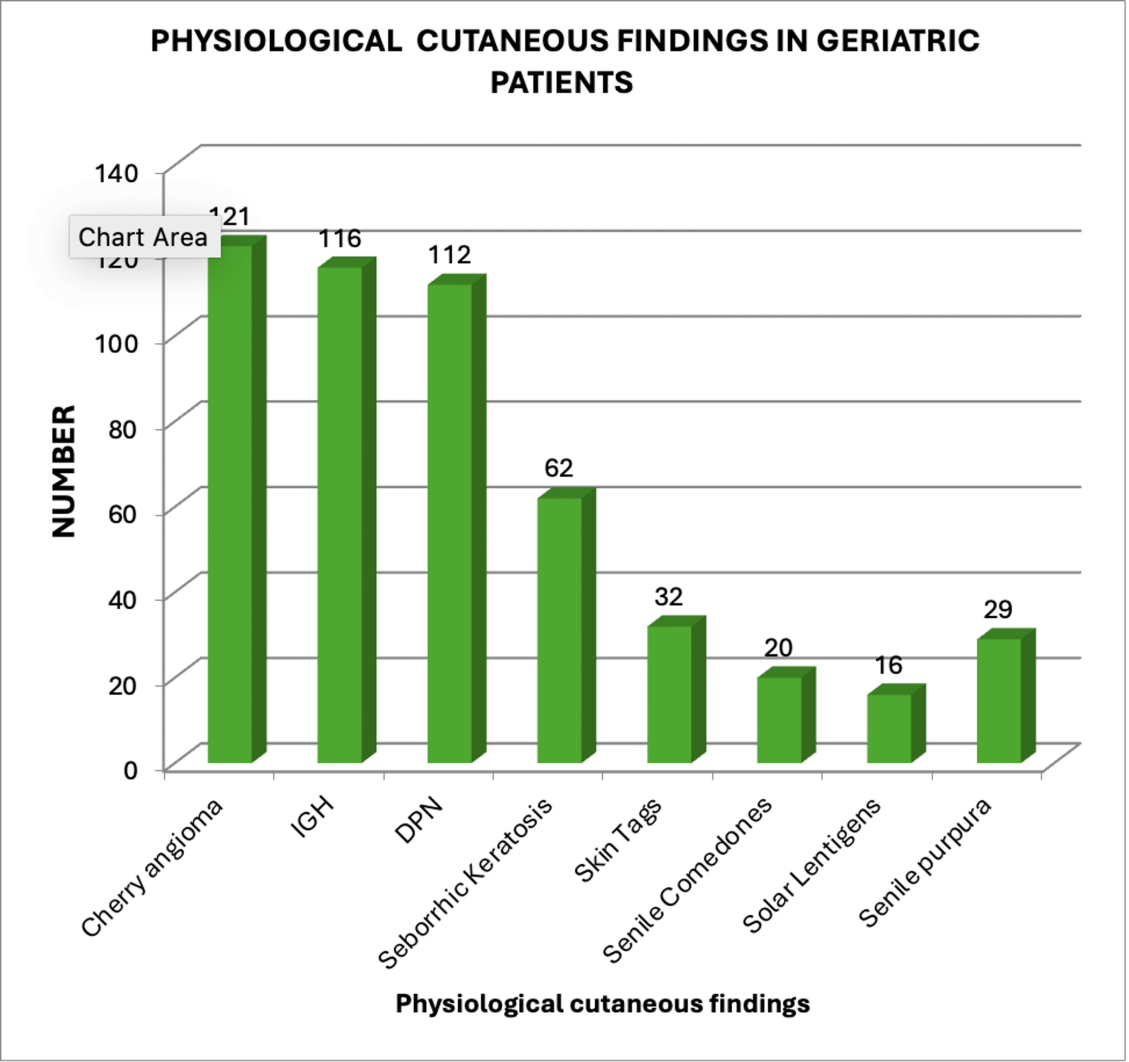
Physiological cutaneous finding in geriatric patients DPN - dermatosis papulosa nigra; IGH - idiopathic guttate hypomelanosis

In this study, physiological cutaneous findings were seen among geriatric patients such as cherry angioma in 121 (25.1%), idiopathic guttate hypomelanosis in 116 (24.06%), dermatosis papulosa nigra in 112 (23.23%), seborrheic keratosis in 62 (12.86%), skin tags in 32 (6.6%), senile comedones in 20 (4.1%), solar lentigines in 16 (3.3%), and senile purpura in 29 (6.0%) (Figure 1).

The distribution of systemic-associated diseases among geriatric patients (N = 482; 282 males, 200 females) highlights gender-specific trends. HTN was the most common condition, with 86 (17.8%) of patients affected. Among them, 75 (15.6%) were already on treatment, and 11 (2.3%) were newly diagnosed. Females had a slightly higher total HTN prevalence (19%) compared to males (17%).

DM affected 50 (10.4%) of patients, with 37 (7.7%) on treatment and 13 (2.7%) newly diagnosed. Males had a higher total DM prevalence (31, 11%) than females (19, 9.5%). Combined HTN and DM were present in 22 (4.6%) of patients, with 19 (3.9%) receiving treatment and three (0.9%) newly diagnosed. This combination was more frequent in males (15, 5.3%) compared to females (seven, 3.5%).

Other systemic conditions, such as bronchial asthma (seven, 1.5%), IHD (four, 0.8%), and pulmonary Koch 5(1%), were less prevalent. Pulmonary Koch was more common in females (four, 2%) than males (one, 0.4%) (Table 3).

**Table 3:** Distribution of systemic associated diseases according to gender among geriatric patients DM - diabetes mellitus; HTN - hypertension; t/t - treatment

Diseases	Total (N = 482)		Male (N = 282)		Female (N = 200)	
	N	%	N	%	N	%
HTN on t/t	75	15.6	40	14.2	35	17.5
Newly diagnosed HTN	11	2.3	8	2.8	3	1.5
Total HTN	86	17.8	48	17.0	38	19
DM on t/t	37	7.7	22	7.8	15	7.5
Newly diagnosed DM	13	2.7	9	3.2	4	2.2
Total DM	50	10.4	31	11.0	19	9.5
HTN + DM on t/t	19	3.9	13	4.6	6	3
Newly diagnosed HTN + DM	3	0.9	2	0.7	1	0.5
Total DM + HTN	22	4.6	15	5.3	7	3.5
Bronchial asthma on t/t	7	1.5	4	1.4	3	1.5
Ischemic heart disease on t/t	4	0.8	3	1.06	1	0.5
Pulmonary Koch on t/t	5	1.0	1	0.4	4	2

The distribution of skin diseases with respect to systemic diseases (Table 4 and Table 5) provides insights into how systemic conditions such as HTN, DM, a combination of HTN and DM (HTN + DM), asthma, pulmonary tuberculosis (PTB), and IHD associated with dermatological conditions among geriatric patients.

**Table 4:** Distribution of skin diseases with respect to the presence of systemic diseases DM - diabetes mellitus; HTN - hypertension; IHD - ischemic heart disease; PTB - pulmonary tuberculosis

Systemic diseases	N = 482		HTN N = 86		DM N = 50		HTN + DM N = 22		Asthma N = 7		PTB N = 5		IHD N = 4	
Skin diseases	N	%	N	%	N	%	N	%	N	%	N	%	N	%
Eczemas	100	20.7	15	17.4	7	14	3	13.6	1	14.3	1	20	-	-
Fungal infections	60	12.4	5	5.8	12	24	7	31.8	-	-	1	20	1	25
Contact dermatitis	50	10.4	6	7.0	2	4	-	-	-	-	1	20	-	-
Papulosquamous disorders	47	9.8	15	17.4	6	12	2	9.1	-	-	1	20	-	-
Viral infection	32	6.6	12	14.0	1	2	1	4.5	1	14.3	-	-	-	-
Xerosis	40	8.3	10	11.6	4	8	1	4.5	-	-	-	-	-	-
Senile pruritus	25	5.1	4	4.6	-	-	-	-	-	-	-	-	2	50
Hansen’s disease	19	3.9	3	3.5	1	2	-	-	1	14.3	-	-	1	25
Photodermatoses	15	3.1	-	-	4	8	1	4.5	-	-	-	-	-	-
Bacterial infections	13	2.7	1	1.2	5	10	-	-	1	14.3	-	-	-	-
Urticaria	12	2.5	1	1.2	-	-	1	4.5	1	14.3	-	-	-	-
Fissured soles	12	2.5	3	3.5	-	-	-	-	-	-	1	20	-	-
Vitiligo	11	2.3	-	-	1	2	1	4.5	-	-	-	-	-	-

**Table 5:** Distribution of skin diseases with respect to the presence of systemic diseases DM - diabetes mellitus; HTN - hypertension; IHD - ischemic heart disease; PTB - pulmonary tuberculosis *p-value given for the chi-square test and for Fisher's exact test when cell values are less than five.

Skin diseases	N = 482		HTN N = 86		DM N = 50		HTN + DM N = 22		Asthma N = 7		PTB N = 5		IHD N = 4	
	N	%	N	%	N	%	N	%	N	%	N	%	N	%
Vesiculobullous disorder	10	2.1	5	5.8	1	2	-	-	-	-	-	-	-	-
Adverse drug reaction	6	1.2	1	1.2	-	-	1	4.5	-	-	-	-	-	-
Pruritus due to systemic diseases	6	1.2	-	-	4	8	1	4.5	1	14.3	-	-	-	-
Scabies	6	1.2	2	2.3	-	-	2	9.0	-	-	-	-	-	-
Malignancy	5	1.0	2	2.3	-		-	-	-	-	-	-	-	- -
Connective tissue disorder	3	0.6	-	-	-	-	-	-	-	-	-	-	-	-
Lichen amyloidosis	3	0.6	-	-	-	-	1	4.5	1	14.3	-	-	-	-
Granuloma annulare	2	0.4	-	-	2	4	-	-	-	-	-	-	-	-
Keloid	2	0.4	1	1.2	-	-	-	-	-	-	-	-	-	- -
Lichen sclerosus et atrophicus	1	0.2	-	-	-	-	-	-	-	-	-	-	-	-
Black hairy tongue	1	0.2	-	-	-	-	-	-	-	-	-	-	-	-
Actinic porokeratosis	1	0.2	-	-	-	-	-	-	-	-	-	-	-	-
p-value *(combined for Tables 4-5)	-	-	<0.001		0.033		<0.001		0.357		0.969		NA	

Table 4 highlights the most prevalent skin diseases. Eczemas were the most common condition, affecting 100 (20.7%) of patients, with notable associations in HTN (15, 17.4%) and DM (seven, 14%). Fungal infections were observed in 60 (12.4%) of cases, with a stronger association in DM (12, 24%) and HTN + DM (seven, 31.8%). Contact dermatitis was seen in 50 (10.4%) patients, primarily associated with HTN (six, 7%) and DM (two, 4%). Papulosquamous disorders affected 47 (49.8%) patients, showing significant prevalence in HTN (15, 17.4%) and DM (six, 12%). Other conditions, such as xerosis, viral infections, and senile pruritus, had varying degrees of prevalence but were less frequently linked to systemic diseases.

Table 5 focuses on less common dermatological conditions. Vesiculobullous disorders (10, 2.1%) were associated with HTN (five, 5.8%), while adverse drug reactions (six, 1.2%) and pruritus due to systemic diseases (six, 1.2%) were associated with HTN + DM and asthma. Conditions like scabies, malignancy, and lichen amyloidosis showed infrequent occurrence, particularly with HTN and PTB. On comparing associations of various systemic diseases, it was observed that HTN, diabetes, and combined HTN and diabetes were significantly associated with diseases such as eczema fungal infections, contact dermatitis, papulosquamous disorder, viral infections, etc.

## Discussion

Aging is an ongoing biological phenomenon. Throughout the aging process, numerous processes, including regenerative ability, chemical detoxification, DNA repair, sensory perception, mechanical protection, and immune response of cellular structures in organs and tissues, deteriorate. The alteration of cellular processes negatively impacts the skin structure, similar to other organs and systems [10]. Considering this, the present study has explored the patterns of dermatoses occurring in the geriatric population with respect to age, gender, as well as the presence of systemic diseases.

Gender-based distribution of dermatological diseases

The prevalence of eczemas, fungal infections, and contact dermatitis showed notable differences between genders. Eczemas, the most prevalent condition, were noted more often in males (23.8%) than in females (16.5%; p = 0.065), which aligns with the study suggesting that male skin physiology may render them more susceptible to such conditions due to variations in barrier function and immune response as per Proksch et al. and Uberoi et al. [11,12]. Conversely, fungal infections were markedly more common in males (15.6%) compared to females (8.0%; p = 0.014). This corresponds with the findings of Gupta et al. and Oliveira et al., which indicated that increased sweat gland activity and occupational exposure in males facilitate fungal infections [13,14].

In contrast, contact dermatitis was more prevalent in females (15.5%) compared to males (6.7%; p < 0.001), possibly attributable to heightened exposure to allergens in cosmetics and home chemicals, as evidenced by research conducted by Diepgen and Weisshaar [15]. Conditions like papulosquamous illnesses, xerosis, and viral infections exhibited no notable gender disparities, indicating equivalent vulnerability in males and females.

The data highlights the need for potential gender-sensitive approaches to dermatological care in geriatric populations.

Age-related distribution of dermatological diseases

Age-related conditions in the present study findings indicated that eczemas were the most common across all age groups, with the highest proportion observed in the 80-89 age group (28.6%; p = 0.508). Though not statistically significant, it may suggest the age-related decline in skin barrier function and immune efficiency [16]. Fungal infections exhibited a slight decrease in the oldest age group (7.1%; p = 0.679), likely attributable to diminished physical activity and restricted exposure.

Contact dermatitis was of a higher prevalence in older age groups, reaching 14.3% (p = 0.248) in the 80-89 age category [17]. Cumulative exposure to irritants as well as allergens over a lifetime may precipitate this phenomenon [18]. Papulosquamous disorders and xerosis showed consistent prevalence across various geriatric age groups, highlighting their chronic nature and the impact of intrinsic factors related to aging. Malignancy was significantly more prevalent in the 70-79 age group (3.0%; p = 0.031), undermining the elevated cancer risk associated with aging. 

Bolognia et al. also found malignancy to be significantly more prevalent in the 70-79 age group (3.0%; p = 0.031) compared to younger cohorts, suggesting increased cumulative UV exposure and the diminished ability of aging skin to repair DNA damage, predisposing individuals to skin cancers [18].

Physiological dermatoses in geriatrics

Physiological cutaneous findings were seen among geriatric patients, providing valuable insights into common dermatological manifestations of aging. The present study found cases of cherry angioma in 25.1%, idiopathic guttate hypomelanosis in 24.06%, dermatosis papulosa nigra in 23.23%, seborrheic keratosis in 12.86%, skin tags in 6.6%, senile comedones in 4.1%, solar lentigines in 3.3%, and senile purpura in 6.0% patients, which aligns with the studies done across the world such as Bolognia et al. reported 25.1% cherry angiomas among elderly [18], Kim et al. reported 20-25% idiopathic guttate hypomelanosis [19]. Researchers such as Leung and Barankin have emphasized the genetic predisposition of dermatosis papulosa nigra, particularly among individuals of African or Asian descent [20]. Findings from a meta-analysis by Sun et al. reported rates of seborrheic keratosis ranging from 10% to 20% [21,22]. These benign epidermal growths are considered a hallmark of aging skin, exacerbated by genetic and environmental factors. Skin tags in geriatric populations have been associated with metabolic syndromes, as noted by Shenoy et al., while senile comedones are linked to follicular changes due to reduced sebaceous gland activity, as described by James et al. [22,23]. Senile purpura reflects dermal thinning and fragility of blood vessels, as commonly reported in geriatric dermatology. Park noted similar rates, contributing the condition to age-related collagen degradation and reduced vascular support [24]. The high prevalence of benign physiological findings emphasizes the fact that differentiating these changes from pathological conditions, especially in geriatric care, is necessary. Regular skin assessments can reassure patients, prevent unnecessary interventions, and identify potential malignancies or systemic associations.

Systemic diseases and their association with dermatological conditions

HTN was observed in 17.8% of the study population and showed a significant association with eczema, which was present in 17.4% of cases. This correlation is consistent with findings by Silverberg et al., which associated systemic inflammation, a characteristic of HTN, with the worsening of eczematous skin conditions [25]. Chronic vascular alterations in hypertensive individuals may compromise the skin’s barrier function, increasing susceptibility to inflammatory disorders.

The research found vesiculobullous disorders in 2.1% of cases, with a correlation to HTN in 5.8% of instances. Kandwal et al. reported similar findings, identifying increased vascular permeability and inflammation as key factors contributing to blistering conditions in hypertensive patients. The results underscore the importance of microvascular damage in the pathology of skin diseases [26].

DM was identified in 10.4% of patients, with a significant 24% prevalence of fungal infections in the diabetic population. This is consistent with research by Gupta et al., which highlighted the impact of hyperglycemia on enhancing fungal growth and compromising immune responses [13]. The high prevalence of fungal infections in patients with concurrent HTN and DM (31.8%) underscores the detrimental effects of these systemic conditions on skin health.

DM was associated with eczemas in 14% of instances, likely due to chronic inflammation and poor glycemic control, which worsen skin barrier dysfunction. Salari et al. also noted an increased risk of inflammatory skin conditions in diabetic patients, attributing this to microvascular complications [27].

Adverse drug reactions (1.2%) and pruritus associated with systemic diseases (1.2%) were primarily observed in patients with both HTN and DM. The results correspond with the study of Chung et al., which demonstrated increased pruritus in patients with numerous chronic conditions due to systemic inflammation and altered cutaneous innervation [28].

Scabies and malignancies, though rare, demonstrated occasional correlations, underscoring their complex nature. Park et al. discovered that scabies in senior populations are associated with immunocompromised conditions, while malignancies may be related to UV exposure and systemic vulnerabilities associated with chronic systemic conditions [24].

The research highlights the necessity of a strategy for the management of elderly patients with systemic diseases. Regular skin evaluations in patients with HTN and DM can promote the early identification and management of related dermatological issues. Targeted interventions, including enhanced glycemic control for diabetics and anti-inflammatory approaches for hypertensive patients, may substantially enhance skin health outcomes.

This study's shortcomings encompass its cross-sectional design, which restricts the capacity to determine causality or monitor temporal changes in dermatological disorders. The sample size and population may not accurately represent all geriatric adults, as the sample is hospital-based, hence diminishing generalizability. Moreover, relevant confounding variables, including lifestyle habits, environmental exposures, and medication usage, were not thoroughly investigated, which may affect the outcomes.

## Conclusions

This study provides a thorough illustration of dermatological disorders in elderly people, their associations with systemic diseases, and age-related alterations. Eczemas, fungal infections, and contact dermatitis were found to be the most common conditions, with significant variations based on gender and age. The findings highlight the significant interaction between systemic diseases, such as HTN and DM, and dermatological symptoms, including eczemas and fungal infections. Physiological cutaneous alterations, such as cherry angiomas and seborrheic keratosis, were frequently noted, highlighting the necessity of differentiating benign age-related disorders from problematic manifestations.

The research emphasizes the importance of a cohesive, interdisciplinary strategy in the diagnosis and treatment of dermatological disorders in the elderly. Customized techniques that take into account age, gender, and overall health can markedly boost clinical outcomes and improve the quality of life for elderly patients.
